# How Microbes Twist Jasmonate Signaling around Their Little Fingers

**DOI:** 10.3390/plants5010009

**Published:** 2016-01-19

**Authors:** Selena Gimenez-Ibanez, Andrea Chini, Roberto Solano

**Affiliations:** Plant Molecular Genetics Department, Centro Nacional de Biotecnología-CSIC (CNB-CSIC), 28049 Madrid, Spain; achini@cnb.csic.es (A.C.); rsolano@cnb.csic.es (R.S.)

**Keywords:** hormonal manipulation, jasmonate, hormone mimics, phytotoxin, coronatine, effectors, JAZ repressors, pathogenesis, symbiosis

## Abstract

Plant immunity relies on a complex network of hormone signaling pathways in which jasmonic acid (JA) plays a central role. Successful microbial pathogens or symbionts have developed strategies to manipulate plant hormone signaling pathways to cause hormonal imbalances for their own benefit. These strategies include the production of plant hormones, phytohormone mimics, or effector proteins that target host components to disrupt hormonal signaling pathways and enhance virulence. Here, we describe the molecular details of the most recent and best-characterized examples of specific JA hormonal manipulation by microbes, which exemplify the ingenious ways by which pathogens can take control over the plant’s hormone signaling network to suppress host immunity.

## 1. Introduction

Agricultural productivity depends on the capacity of plants to adapt to changing environmental conditions. In nature, plants live in complex environments in which they intimately interact with a broad range of microbial pathogens with different infection strategies. To defend themselves, plants have evolved sophisticated strategies to perceive their attacker during the infection process and to translate this perception into an effective immune response. Recognition of highly conserved microbe-associated molecular patterns (MAMPs) by host cell transmembrane pattern recognition receptors (PRRs) leads to MAMP-triggered immunity (MTI) that restricts pathogen growth [1]. To overcome such lines of defense, adapted pathogens deliver phytotoxins and virulence molecules called effector proteins into the plant cell to promote pathogenesis in a process known as effector-triggered susceptibility (ETS) [1]. Inside the plant cell, phytotoxins and effectors target host molecules to subvert the host cell physiology and disrupt defenses. However, despite the fact that elucidating effector action is essential to understanding bacterial pathogenesis, the molecular function and host targets of the vast majority of effectors remain largely unknown.

Plant immunity relies on a complex network of hormone signaling pathways in which jasmonic acid (JA) and salicylic acid (SA) play a central role [2,3]. Both molecules control defense responses to different types of microbes and thus, they orchestrate a different and complex transcriptional reprogramming response that eventually leads to plant resistance. The JA pathway is primarily induced by and effective in mediating resistance against herbivores and necrotrophic pathogens such as *Botrytis cinerea*, whereas the SA pathway is primarily induced by and effective in mediating resistance against biotrophic and hemi-biotrophic pathogens such as *Pseudomonas syringae* [3]. JA and SA defense pathways generally antagonize each other and thus, elevated resistance against necrotrophs is often correlated with increased susceptibility to biotrophs and *vice versa* [4]. JAs play a key role in plant survival by contributing to adaptation to biotic and abiotic stresses and modulating many physiological and developmental agricultural traits such as root growth and fertility [5]. In the complex picture of hormonal plant immunity, other hormones such as abscisic acid (ABA), auxins, brassinosteroids (BRs), cytokinins (CKs), ethylene (ET), gibberellic acid (GAs), and oxylipins (other than JA) function as modulators of the plant immune signaling network as well, fine-tuning the hormonal balances to optimize resistance to the invading organism [2,6,7]. Therefore, the collective contribution of hormones during plant-pathogen interactions is crucial to establish the robust immune transcriptional program against the specific infection strategy of invading attackers.

In the last decade, a combination of genetic, molecular, and biochemical analyses have dissected the core JA signal transduction components linking JA synthesis to hormone-induced changes in gene expression. Among all JAs found in nature, (+)-7-iso-JA-l-Ile (JA-Ile) is the molecularly active form of the JA hormone [8]. JA-Ile is perceived through a co-receptor complex formed by the F-box protein CORONATINE-INSENSITIVE 1 (COI1) and JASMONATE ZIM DOMAIN (JAZ) proteins [9,10,11,12]. COI1 controls the turnover of the JAZ co-receptors in response to JA-Ile. The JAZ family is composed of 12 canonical members in *Arabidopsis* and an atypical repressor, JAZ13 [13,14]. JAZ proteins are repressors of the JA-signaling transcription factors (TFs), such as the basic helix-loop-helix (bHLH) MYC2, MYC3 and MYC4, that control JA-regulated genes [9,10,11,15,16,17]. To initiate transcription, MYCs physically interacts with the MEDIATOR25 (MED25) subunit of the eukaryotic Mediator complex [17,18,19,20]. This complex recruits to the MYCs-bound promoter all of the general transcription factors (GTFs) and RNA polymerase II machinery required to initiate JA transcriptional reprograming [19,20]. Repression of TFs by JAZ involves the competitive inhibition of MYC interaction with MED25 by the Jas domain of JAZs [17]. Repression also requires the recruitment of the general co-repressor TOPLESS (TPL) and TPL-related proteins through the NOVEL INTERACTOR OF JAZ (NINJA) adaptor, which are supposed to lock chromatin by histone deacetylation [21]. Under stress conditions, JA-Ile promotes the formation of JAZ-COI1 complexes, triggering JAZ ubiquitination and subsequent degradation via the 26S proteasome [9,10,11]. This leads to de-repression of the TFs that initiate the transcription of JA-dependent genes [22].

Pathogens have evolved capabilities to manipulate or subvert plant hormone signaling pathways to cause hormonal imbalances for their own benefit. Microbes enhance their virulence by producing plant hormones, phytohormone mimics, or injecting into eukaryotic plant cells an arsenal of virulence effector proteins that target hormonal components to evade or disrupt hormonal signaling pathways and/or crosstalk [23]. Here, we describe the molecular details of the best-characterized examples of specific JA hormonal manipulation by microbes, which exemplify the ingenious ways by which pathogens can take control over the plant’s hormone signaling network to suppress host immunity.

## 2. Production of JA Phytohormones and Mimics by Pathogens

**Figure 1:**
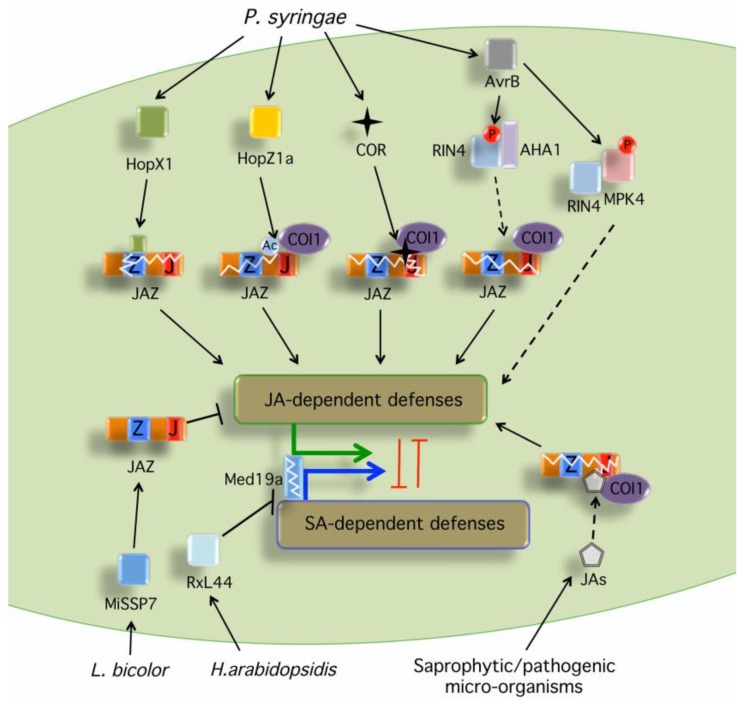
Phytotoxins and microbial effectors targeting the JA signaling components. Several microbes produce JA-Ile precursors or the JA-Ile mimic coronatine that are perceived by the JA-Ile receptor complex and induce degradation of JAZ repressors (see Table 1 for specific examples). HopX1 from *P. syringae* pv. *tabaci* (*Pta*) 11528 encodes a cysteine protease that associates with JAZ proteins induces their degradation in a proteasome- and COI1-independent manner, likely via its cysteine protease activity [56]. HopZ1a from *P. syringae* pv. *syringae* (*Pss*) strain A2 encodes a cysteine protease/acetyltransferase that acetylates several JAZ proteins and induces their degradation through an undefined mechanism that is dependent on COI1 [57]. AvrB interacts with [71] and phosphorylates MPK4, which triggers activation of JA signaling [58]. AvrB also induces the degradation of multiple JAZ proteins by positively regulating the PM H+-ATPase AHA1 to enhance bacterial penetration and virulence through RIN4 [59]. The ectomycorrhizal MiSSP7 effector interacts with PtJAZ6 and protects PtJAZ6 from JA-induced degradation, attenuating JA-dependent host defenses to promote fungal colonization and mutualism [61]. The oomycete downy mildew effector RxL44 directly interacts with the mediator subunit MED19a (Mediator19a), resulting in the proteasome-mediated degradation of MED19a, which shifts the balance of defense gene transcription from SA-responsive to JA/ET-mediated defense, enhancing susceptibility to biotrophs [60]. In all cases, degradation of JAZs leads to de-repression of the TFs that initiate the activation of JA-dependent gene expression, suppression of SA responses and plant susceptibility. Black arrows indicate activation of the indicated hormonal pathway. Black bars indicate inhibition of the indicated hormonal pathway. Dashed arrows denote indirect or unclear mechanism leading to the activation of the indicated hormonal pathway. A circled Ac indicates acetylation. A circled P indicates phosphorylation.

Pathogens are capable of synthesizing phytohormones and phytohormone mimics to take control of the host immune system by inducing hormonal imbalances (Table 1). Probably, the best-studied example corresponds to coronatine (COR), a mimic of the bioactive JA-Ile hormone, produced by some strains of *P. syringae* [8,24,25]. COR, as JA-Ile, is perceived through the COI1/JAZ co-receptor complex, activating JA-dependent responses that, in turn, inhibit SA-dependent defenses required for *P. syringae* resistance (Figure 1) [5,26]. Remarkably, COR is more active than the JA-Ile plant hormone itself in triggering the COI1-JAZ complex formation and subsequent JAZ degradation, indicating that COR acts as a potent and highly specific mimic of JA-Ile perception in plants [8,27]. The functional similarity between COR and JA-Ile has been further demonstrated by the three-dimensional structural analysis of each molecule in association with a COI1 receptor complex [11], and by the rational design of COR-based antagonists of JA-Ile perception that block the COI1-JAZ co-receptor [28]. Among the twelve canonical JAZs, eight genes are induced in *Arabidopsis* during *P. syringae* infection in a COR-dependent manner [29]. Of note, only loss-of-function mutants of *JAZ10* are more susceptible to *P. syringae*, indicating that JAZ10 is a negative regulator of both JA signaling and disease development [29]. In combination with *jaz10,* the *jaz5* knockout (KO) enhances chlorotic symptoms and (moderately) bacterial growth, indicating that JAZ5 and JAZ10 act co-operatively to restrict *P. syringae* growth during bacterial pathogenesis and are targeted by COR [30].

In plants, COR acts as a multifunctional suppressor of defense responses and *P. syringae* mutants unable to produce COR show reduced virulence [24,31]. COR contributes to disease symptomatology by inducing chlorotic lesions [24,32,33], facilitates bacterial entry into the plant host by stimulating the re-opening of stomata after microbial-triggered stomatal closure [34,35,36], promotes bacterial growth by inhibiting SA-dependent defenses required for *P. syringae* resistance through the activation of its antagonistic JA pathway [37,38] and suppresses plant cell wall defense through perturbation of secondary metabolism [31]. Analyses of both plant and bacterial mutants indicate that COR elicits the JA pathway to repress SA-signaling, and that suppression of SA responses is a necessary component of the virulence action of COR [2,32,33,39,40,41]. A mechanism for suppression of SA-signaling by COR occurs though MYC2 TF that binds to the promoter and triggers the expression of the three NAC (petunia NAM and *Arabidopsis* ATAF1, ATAF2, and CUC2) *ANAC19, ANAC55* and *ANAC72* TFs [39]. These ANACs repress expression of genes involved in SA-biosynthesis, such as Isochorismate Synthase 2 (*SID2*), and induce *BSMT1*, a benzoic/salicylic acid carboxyl methyltransferase that reduces the biologically active pool of SA. Consequently, COR suppresses SA-mediated plant immunity against the bacteria, inducing stomatal reopening and bacterial propagation in both local and systemic tissues [39]. Remarkably, closely related NAC TFs, JA2 (Jasmonic Acid 2) and JA2L (JA2-like) differentially regulate stomatal closure and reopening during *Pseudomonas* attack in tomato [42], further indicating that the function of NAC TFs is conserved among different plant species.

**Table 1:** Microorganisms able to synthetize jasmonates (JAs) and JA-mimics.

Micro-Organism	Class	Molecules	Reference
*Pseudomonas syringae*	Pathogenic bacteria	coronatine	[25]
*Lasiodiplodia theobromae*	Pathogenic fungus	JA	[43]
*Gibberella fujikuroi*	Pathogenic fungus	JA-Ile and other jasmonates	[44]
*Collybia confluens*	Saprophitic fungus	JA and 7-iso-JA	[45]
*Collybia dryophila*	Saprophitic fungus	JA and 7-iso-JA	[45]
*Coprinus alkalinus*	Saprophitic fungus	JA and 7-iso-JA	[45]
*Coprinus cinereus*	Saprophitic fungus	JA and 7-iso-JA	[45]
*Mycena tintinabulum*	Saprophitic fungus	JA and 7-iso-JA	[45]
*Phellinus laevigatus*	Saprophitic fungus	JA and 7-iso-JA	[45]
*Trametes versicolor*	Saprophitic fungus	JA and 7-iso-JA	[45]
*Pisolithus tinctorius*	Ectomyccorhizal fungus	JA and 7-iso-JA	[46]
*Fusarium oxysporum*	Pathogenic fungus	JA, JA-Ile, 9,10-dihydro-JA and other jasmonates	[47,48,49]
*Diplodia gossypina*	Pathogenic fungus	JA and Me-JA	[50]
Strains SF2, SF3 and SF4	Endophytic bacteria	OPDA and other jasmonates	[51]
*Magnaporthe oryzae*	Pathogenic fungus	JA, Me-JA and 12OH-JA	[52]

A COI1-independent effect of COR has also been reported [31]. COR seems to suppress an SA-independent pathway contributing to callose deposition, a hallmark of plant resistance, by reducing accumulation of an indole glucosinolate via PEN2 (penetration 2)-dependent pathway [31]. However, this COI1-independent effect of COR awaits further confirmation due to the use of weak alleles of *coi1.* Thus, acquisition of COR by *Pseudomonas* pathogens has been of tremendous adaptive importance during host-pathogen evolution to manipulate the host hormonal network to promote susceptibility.

Several fungi such as *Fusarium oxysporum* (*F. oxysporum*) and *Lasiodiplodia theobromae* (*L. theobromae*) are also capable of producing bioactive JAs among others (Table 1, Figure 1) [23,43,44,45,46,47,50,51,53,54]. For example, the tomato infecting fungus *F. oxysporum* forma specialis (f. sp) *lycopersici* produces JAs through the specific lipoxygenase enzyme 13*S*-LOX, conserved with plant LOXs [48]. In addition, *F. oxysporum* f. sp. *conglutinans* and *F. oxysporum* f. sp. *matthioli*, which infect roots of *Arabidopsis*, produce JA-Ile and JA-Leu, respectively, in culture filtrates [49]. Consequently, JA-insensitivity suppresses infection by these *F. oxysporum* strains that produce detectable JA-Ile levels [49]. This indicates that several fungi have also evolved to produce JAs as a virulence strategy to promote infection [49].

On the other hand, some pathogens have evolved the competence to detoxify or inactivate compounds required for immunity including JAs [8,55]. For example, hydroxylation of JAs is a common catabolic mechanism contributing to switch-off JA signaling but producing derivatives that may have other functions [55]. The rice blast fungus *Magnaporthe oryzae* (*M. oryzae*) produces an antibiotic biosynthesis monooxygenase (Abm) that converts endogenous free JA into hydroxylated JA (12OH-JA) [52]. Both Abm and 12OH-JA are secreted during host penetration and help evade plant defenses [52]. Loss of Abm in *M. oryzae* leads to accumulation of plant-produced methyl (Me)JA blocking the invasive growth of the fungi, whereas exogenously added 12OH-JA markedly attenuates abmΔ-induced immunity in rice. Therefore, Abm might have a dual role, on one hand reducing the free JA, and therefore plant defenses, and, on the other hand, producing 12OH-JA, which facilitates *M. oryzae* infection [52].

## 3. Effector-Mediated Manipulation of JA Signaling Pathway by Bacterial and Oomycete Plant Pathogens

Bacterial pathogens such as *P. syringae* have evolved specific effectors to disrupt hormonal equilibrium by targeting JA signaling and/or JA-SA balance in the plant cell (Table 2, Figure 1) [6,56,57,58,59].

The *Pseudomonas* effectors HopX1 and HopZ1a activate the JA signaling pathway by targeting the JAZ repressors [56,57]. HopX1 from *P. syringae* pv. *tabaci* (*Pta*) 11528 encodes a cysteine protease that associates with JAZ proteins via its conserved ZIM domain and induces JAZ degradation in a proteasome- and COI1-independent manner, likely via its cysteine protease activity [56]. Ectopic expression of HopX1 in *Arabidopsis* induces the expression of JA-dependent genes and represses SA signaling. Delivery of HopX1 by the natural TTSS (Type Three Secretion System) of *Pseudomonas* partially re-opens stomata and promotes susceptibility during the infection process. Since *Pta* 11528 does not produce COR, HopX1 may have evolved as an alternative evolutionary strategy to manipulate core regulators of JA signaling.

**Table 2:** Microbial effectors targeting JA-mediated defenses.

Effector	Organism	Plant Target	Mode of Action	Reference
AvrB (MPK4)	*P. syringae*	RIN4/MPK4	Activates JA defenses targeting	[58]
			MPK4/RIN4/HSP90/RAR1 complex	
AvrB (AHA1)	*P. syringae*	RIN4/AHA1	Activates JA defenses promoting AHA1-dependent	[59]
			COI1/JAZ interaction and JAZ degradation	
HopZ1a	*P. syringae*	JAZs	Activates JA defenses promoting COI1-dependent	[57]
			degradation of the JAZ repressors	
HopX1	*P. syringae*	JAZs	Activates JA defenses promoting COI1-independent	[56]
			degradation of the JAZ repressors	
RxL44	*H. arabidopsidis*	MED19a	Induces MED19a degradation shifting the balance	[60]
			from SA- to JA/ET-mediated defense	
MiSSP7	*L. bicolor*	JAZs	Suppresses JA defenses stabilizing the JAZs	[61]
SSITL	*S. sclerotiorum*	unkown	Suppresses JA defenses	[62]
Exopolysaccharide	*B. cinerea*	unkown	Activates SA pathway to suppress JA defenses	[63]
Abm	*M. oryzae*	JA or unkown	Suppresses JA defenses by convertion of fungal JA	[52]
			into 12OH-JA	

HopZ1a from *P. syringae* pv. *syringae* (*Pss*) strain A2 encodes a cysteine protease/acetyltransferase that acetylates several JAZ proteins interacting with their conserved Jas domain [57]. HopZ1a-mediated acetylation induces JAZ1 degradation through an undefined mechanism that is dependent on COI1, leading to the activation of JA-dependent gene expression, suppression of SA responses and plant susceptibility (Figure 1) [57]. Interestingly, this suggests that post-translational modifications of the Jas motif of JAZ1 may modulate the 26S proteasome degradation triggered by COI1 in the absence of the hormone. Indeed, post-translational modifications, including acetylation, have been shown to induce or repress proteasomal degradation [64]. Recently, the threonine residue T346 was identified as the main auto-acetylation site of HopZ1a by mass spectrometry, whereas two additional neighboring serine residues (S349 and S351) are required for the acetyltransferase activity of HopZ1a *in vitro* and indispensable for the virulence function of HopZ1a *in planta* [65,66]. These serine residues are important for the enzymatic activity of HopZ1a and are indispensable for inositol hexakisphosphate (IP6)-induced activation of the effector in eukaryotic hosts [65]. The absence of IP6 in bacteria suggests that the enzymatic activity of this effector is activated after it is delivered into the host. Interestingly, previous structural studies have revealed that interaction of the COI1/JAZ co-receptor with its JA-Ile ligand requires an inositol pentakisphosphate (IP5) cofactor [11]. Further investigations are needed to determine how HopZ1a-mediated acetylation of JAZs could facilitate their COI1-dependent degradation and whether this effector mimics a still unknown host post-translational mechanism controlling the activity of the JA receptor complex.

Besides the JA receptor, additional bacterial effectors affect plant hormonal equilibrium targeting other JA signaling components. The kinase-like AvrB effector was among the first effectors shown to promote JA signaling [58,67]. AvrB induces JA signaling by at least two different mechanisms, both dependent on the AvrB-interacting plasma membrane (PM)-associated protein RIN4 (RPM1-interacting) [68,69]. On one hand, AvrB interacts with and phosphorylates MPK4, a mitogen-activated protein kinase that acts as a positive regulator of JA signaling (Figure 1) [58]. AvrB is unable to elicit the expression of the plant defensin *PDF1.2* in *mpk4* mutant plants and when using an *avrB* mutant unable to phosphorylate MPK4, suggesting that phosphorylation of MPK4 is required for AvrB-mediated promotion of JA signaling [58]. Notably, overexpression of AvrB in the *coi1* mutant background fails to enhance susceptibility to *P. syringae* [67], indicating that the AvrB-mediated defense suppression requires the JA receptor COI1 and canonical components of the JA signaling pathway.

On the other hand, AvrB also induces stomatal opening by positively regulating the PM H+-ATPase AHA1 to enhance bacterial penetration and virulence in a RIN4-dependent manner (Figure 1) [59,70,71]. RIN4 directly interacts with AHA1 and the closely related AHA2, acting as a regulator of the proton pump required for stomatal dynamics, likely to inhibit the entry of bacterial pathogens into the plant leaf during infection [71,72]. AvrB is able to interact with RIN4 and promote its phosphorylation [69,73,74], and RIN4 phosphorylation mimics show enhanced H+-ATPase activity and inhibited immune responses activated upon microbial perception in *Arabidopsis* [70]. Congruently, transgenic expression of AvrB enhances H+-ATPase activity of AHA1, and bacterially delivered AvrB induces stomatal opening and enhances infection in a RIN4-dependent manner, demonstrating that the RIN4-AHA1 pathway is exploited by AvrB for virulence [59]. Interestingly, AHA1 promotes the interaction between the JA receptor COI1 and JAZ proteins, activating JA signaling. Similarly, AvrB also induces the COI1-JAZ9 interaction and the degradation of multiple JAZ proteins [59]. Thus, AvrB-induced stomatal opening and virulence requires the canonical JA signaling pathway, which involves COI1, JAZs and also previously described ANAC19, ANAC55 and ANAC72 TFs [39,59]. The biochemical mechanism by which the PM AHA1 activity induces nuclear COI1-JAZ interaction remains unclear. One hypothesis speculates that activation of PM H+-ATPase could induce post-translational modification of JAZs leading to JAZ degradation as, for example, the acetyltransferase activity of the bacterial effector HopZ1a [57,59]. Alternatively, the activation of PM H+-ATPase could induce the generation of JAs or unknown signal molecules/cofactors such as inositol pentakisphosphate IP5 to potentiate the assembly of the COI1-JAZ complex [59]. In this context, a recent report suggests that inositol pyrophosphate IP8 may also be a critical cofactor in COI1-JAZ complex formation as *Arabidopsis* plants lacking VIH2, a diphosphoinositol pentakisphosphate kinase (PPIP5K) regulating the biosynthesis of IP8, are compromised in JA dependent responses [75]. In summary, these findings uncover a novel pathway exploited by AvrB that acts upstream of COI1 to regulate JA signaling and stomatal opening.

The nuclear effector RxL44 encoded by the oomycete downy mildew pathogen *Hyaloperonospora arabidopsidis* directly interacts with the mediator subunit MED19a (Mediator19a), resulting in the proteasome-mediated degradation of MED19a (Figure 1) [60]. The Mediator complex acts as a molecular bridge between transcriptional regulators and RNA polymerase II at the transcription start site, and has emerged as a key regulator of plant immunity [76]. MED25, another component of the Mediator complex, interacts with several TFs including MYC2 and MYC3, acting as an integrative hub for the regulation of JA-responsive gene expression [17,18]). RxL44-induced MED19a degradation shifts the balance of defense gene transcription from SA-responsive to JA/ET-mediated defense, enhancing susceptibility to biotrophs. In contrast with RxL44, plants overexpressing *MED19a* show significant higher levels of SA defenses [60]. Thus, RxL44 enhances susceptibility to biotrophs by attenuating SA-dependent gene expression.

Finally, recent data indicates that independently evolved virulence effectors converge onto hubs in a plant immune system network [77,78]. High throughput systematic protein-protein interaction approaches between proteins from the reference plant *Arabidopsis thaliana* and effector proteins from the bacteria *P. syringae*, the oomycete *Hyaloperonospora arabidopsidis* and the ascomycete *Golovinomyces orontii* pathogens, has allowed to develop a large-scale map of physical interactions [77,78]. These experiments yielded a map of more than 6000 interactions, resulting in a network that identifies host proteins onto which intraspecies and interspecies pathogen effectors converge [77,78]. Pathogens from different kingdoms deploy independently evolved virulence effectors that interact with a set of highly connected cellular hubs to facilitate their diverse life-cycle strategies, showing an interspecies effector convergence onto shared host proteins. Remarkably, some of the most significantly targeted plant hub proteins by effectors include immune- and hormone-related proteins such as JAZ3 and MED19a among others. Thus, it is tempting to speculate that the understanding of how effector molecules from multiple pathogens hijack hormonal pathways to promote disease is just beginning. Altogether, these examples indicate that the JA receptor complex stands as a major hub/target in the arms race between plants and pathogens.

## 4. Effector-Mediated Manipulation of JA Signaling Pathway by Necrotrophic Fungi

Compared to our knowledge on bacterial and oomycete effectors, the molecular mechanisms used by necrotrophic fungi to manipulate JA signaling are still very poorly understood. Infection by necrotrophs primarily relies on the production of nonspecific toxins and cell wall degrading enzymes. However, several examples suggest that this type of pathogens also manipulate hormonal pathways. The necrotrophic fungi *B. cinerea* secretes an exopolysaccharide (EPS) potentially acting as an elicitor of the SA pathway to suppress JA defenses in tomato plants (Table 2) [63]. In another recent example, the necrotrophic fungal pathogen *Sclerotinia sclerotiorum* secretes an effector-like protein called SSITL (sclerotinia sclerotiorum integrin-like) that suppresses JA-dependent defenses [62]. SSITL retains the conserved solvent-exposed Arg-Gly-Asp (RGD)-like motif of plant integrin-like proteins, such as NDR1 (nonrace-specific disease resistance1) involved in defense signaling; therefore suggesting that SSITL mimics the activity of this type of proteins [62]. Further studies are still needed to understand at the molecular level how these molecules act inside plant cells.

## 5. Effector-Mediated Modulation of JA Signaling by Beneficial Microbes

Beneficial microbes establish long-term relationships with their host plants to fulfill their life cycles [79]. Ectomycorrhizal fungi (EM), such as *Laccaria bicolor*, support host growth by providing growth-limiting nutrients to their plant host through a mutualistic symbiotic relationship within the plant roots [80]. In order to develop within the host and feed on living cells, they need to contend with the defense mechanisms of the plant, and fungal secreted effectors may facilitate this process. The mycorrhizal induced small secreted protein 7 (MiSSP7) is encoded by the most highly symbiosis-upregulated gene of the EM *L. bicolor*. MiSSP7 is an effector protein indispensable for the establishment of mutualism with their host plants [80]. Upon perception of diffusible signals from the Populus roots, the fungus secretes MiSSP7 that is imported into the plant cell via phosphatidylinositol-3-phosphate-mediated endocytosis, and targeted to the plant nucleus (Table 2, Figure 1) [61,80]. In the nucleus, MiSSP7 interacts with the JA-repressor PtJAZ6 and protects PtJAZ6 from JA-induced degradation. Consistently, the symbiotic activity of MiSSP7 can be replaced by either inhibiting JA signaling *in planta* or through transgenic overexpression of the *PtJAZ6* gene [61]. This indicates that MiSSP7 is able to inhibit the JA-dependent host defenses stabilizing JAZ repressors to promote fungal colonization and mutualism. Thus, beneficial microbes also evolved effectors that are delivered inside the host plant cell to manipulate hormonal signaling, and thus can be considered symbiotic effectors.

## 6. Conclusions

The importance of signaling molecules in plant immunity is a well-established notion in plant biology and the molecular mechanisms regulating the phytohormone-mediated defenses have been extensively studied in the last decades. In this context, the role of hormones in plant immunity is further highlighted by the increasing number of pathogens producing phytohormones or phytohormone mimics and by the identification of a plethora of microbial effectors targeting hormonal pathways to promote disease or establish beneficial interactions. Of note, virulence activities of effectors on specific hormonal components can lead to the identification of novel mechanisms of host hormonal regulation unknown to date. In addition, an improved mechanistic understanding of how pathogenic toxins and effectors manipulate host hormonal components will be instrumental in identifying new defensive hubs and to develop new plant varieties with improved resistance to pathogens.
